# Evaluation of Self-Reported Oral Health Attitudes and Behavior of Dental Students in Antalya, Turkey

**DOI:** 10.7759/cureus.44387

**Published:** 2023-08-30

**Authors:** Koray Surme, Hayri Akman

**Affiliations:** 1 Department of Pediatric Dentistry, Alanya Alaaddin Keykubat University, Antalya, TUR

**Keywords:** hu-dbi, behavior, attitude, oral health, dental student

## Abstract

Background

Dental students are expected to play a critical role in the oral health education of society as future providers of dental care, and their attitudes on this subject have a possible impact on the awareness of patients regarding the importance of preventing oral diseases and improving oral health. This study aimed to evaluate the oral health attitudes and behavior of dental students.

Methodology

The Turkish version of the Hiroshima University Dental Behavior Inventory (HU-DBI) questionnaire regarding oral health attitudes and behaviors with an additional nine questions was distributed among 250 native Turkish-speaking dental students at the Faculty of Dentistry in Antalya, Turkey. The validity of the nine questions added to the original questionnaire was evaluated by expert opinion and a pretest method. A self-administered questionnaire was used for data collection. The Mann-Whitney U test and chi-square test were used for statistical analysis, and the statistical significance level was set at p-values <0.05.

Results

The mean age of the 250 students in the study was 20.96 ± 1.66 years. In total, 157 (62.8%) students were preclinical (first, second, and third year) dentistry students, and 93 (37.2%) students were clinical (fourth and fifth year) dentistry students. The mean HU-DBI score of all students was 5.95 ± 1.65, and the mean HU-DBI score of clinical students (6.42 ± 1.65) was significantly (p < 0.05) higher than that of preclinical students (5.67 ± 1.59). The mean HU-DBI score of male and female students was 5.63 ± 1.55 and 6.24 ± 1.69, respectively, and this difference was statistically significant (p < 0.05). The study showed significant improvement in the behavior and attitude regarding the oral health of the clinical dental students compared with the preclinical dental students.

Conclusions

Among dental students, clinical students and female students had generally better attitudes and behaviors about oral health self-care compared to preclinical students and male students, respectively.

## Introduction

Oral diseases, especially caries development and periodontal problems, which are prevalent chronic conditions, can be influenced by comorbid systemic diseases or contribute to an elevated likelihood of disease progression [1]. The most effective and accepted method for preventing oral diseases is to maintain good oral health, which requires the use of toothbrushes and toothpaste, as well as interdental aids such as dental floss and other oral care products [2]. The maintenance of optimal oral hygiene and the acquisition of adequate knowledge regarding one’s oral health and care are of utmost significance [3].

The attitudes and behaviors exhibited by oral healthcare professionals in relation to their own personal oral healthcare habits have the potential to serve as an indicator of their understanding of the importance of oral health-promoting procedures. Furthermore, these attitudes and behaviors may play a role in improving the overall oral health of individuals [4]. Dental students, who will work as oral healthcare professionals in the future, have a critical role to play in promoting public oral health awareness [5]. As they are expected to set an example for positive oral health behavior among other college students, their patients, family members, and acquaintances, they need to develop their understanding and commitment to maintaining oral health [6,7].

Makoto Kawamura developed the Hiroshima University Dental Behavioural Inventory (HU-DBI) in 1988 as a psychometric tool to evaluate dental students’ understanding of, attitudes toward, and behaviors related to oral health [8]. The HU-DBI was subjected to demanding check procedures to evaluate its internal and external validity and has demonstrated strong psychometric features [9,10]. Hence, it is widely employed in numerous research studies focusing on dental students in various nations and can pave the way for the development of current programs aimed at improving the attitudes and behaviors of dental students [11-14]. In addition, differences in oral health-related attitudes and behaviors between male and female dental students were assessed with the HU-DBI questionnaire [12,13].

Dental students have the unique opportunity in their educational environment to inspire individuals and communities toward healthy oral self-care. Positive attitudes toward oral health promotion should be developed specifically during student life, not during the rest of life [15]. Numerous studies have used the HU-DBI questionnaire to evaluate variations in oral health behaviors among dental students across different countries owing to the various curricula dental schools offer and the prevalent cultural heterogeneity among them [14,16,17]. Additionally, Cortes et al. (2002) demonstrated that dental students’ attitudes and behaviors toward oral health improve with their level of education [18]. Undergraduate dental education in Turkey consists of a total of five academic years, and although the curricula of the faculties vary, they generally include preclinical (years one, two, and three) years, which include basic medical and dental science courses and laboratory applications, and clinical (years four and five) years, which include clinically oriented courses and on-patient practices [19]. Although clinical dental students’ attitudes and behaviors toward oral health, especially oral hygiene practices, have significantly improved [17], few studies have examined the attitudes and behaviors pertaining to oral health among dental preclinical and clinical students within the Turkish population [6,20]. In this study, a modified HU-DBI questionnaire consisting of nine additional questions was used, based on previous studies, to evaluate the oral health attitudes and behaviors of students in detail (risk factors, impact of education, and oral hygiene practices), unlike the previous studies using the original HU-DBI questionnaire in Turkey [12,21,22]. The objective of this research was to evaluate the variations in self-reported attitudes and behaviors toward oral health among dental students of different academic levels and genders in Antalya, Turkey, utilizing the modified HU-DBI instrument.

## Materials and methods

Sample size calculation

The target population of this study was the students actively enrolled in Alanya Alaaddin Keykubat University Faculty of Dentistry in the 2022-2023 academic year, and the total number of students was 388. Based on this population size, Raosoft Software was used to calculate the sample size with a 95% confidence interval and 5% margin of error by estimating 50% as the response distribution. The minimum sample size calculated by the software was 194. The sample size was set at 250 to prevent possible problems in the study.

Study design and population

A total of 250 native, Turkish-speaking, undergraduate students from five academic years of Alanya Alaaddin Keykubat University Faculty of Dentistry in the academic year 2022-2023 were included in this questionnaire-based, cross-sectional study conducted between November 4 and November 11, 2022. Alanya Alaaddin Keykubat University Faculty of Dentistry is based in the city of Antalya, located on the southern coast of Turkey in the Mediterranean region. Students in the first, second, and third academic years were considered preclinical students, and students in the fourth and fifth academic years were considered clinical students [19].

Study tool

In this study, a modified HU-DBI questionnaire was employed to measure the oral health attitudes and behavior of preclinical and clinical undergraduate dental students. The HU-DBI questionnaire consists of 20 questions related to oral and dental health and tooth brushing habits, with all questions having a two-point response system of agree/disagree. In a previous study among Turkish dental students, a precisely translated Turkish version of the HU-DBI questionnaire was used [23]. In this study, in addition to the 20 questions in the Turkish-translated HU-DBI questionnaire, a modified questionnaire consisting of a total of 29 questions was developed by adding nine new questions based on current studies evaluating the attitudes and behaviors of dental students regarding oral health [12,21,22]. Two different experts from the faculty of dentistry with more than five years of experience were consulted for their recommendations on the nine newly added questions, and the pre-final version of the questionnaire was developed. A pre-test was conducted to obtain information about the feasibility of using the questionnaire and to assess the ease of understanding. The questionnaire was administered to a total of 30 participants, six from each academic year not included in the study. After completing the questionnaire, they were invited to comment on any difficulties in an interview. After the feedback, the final version of the questionnaire was developed.

The evaluation of the questionnaires was based on the method used in the evaluation of HU-DBI questionnaires (Table 1). General oral and dental health and oral care habits scores were calculated by evaluating the answers given to 12 questions. A total score was calculated by assigning one point for agree answers to questions 4, 9, 11, 12, 16, 19 and one point for disagree answers to questions 2, 6, 8, 10, 14, 15. The maximum score was 12, with higher scores indicating better oral health behaviors.

**Table 1:** Items of the modified HU-DBI. *: (A) indicates 1 point for the answer “Agree” to this item and (D) indicates 1 point for the answer “Disagree” to this item. HU-DBI: Hiroshima University Dental Behavior Inventory

Item No	Item
1	I don’t worry much about visiting the dentist
2	My gums tend to bleed when I brush my teeth (D)
3	I worry about the color of my teeth
4	I have noticed some white sticky deposits on my teeth (A)
5	I use a child-sized toothbrush
6	I think that I cannot help having false teeth when I am old (D)
7	I am bothered by the color of my gums
8	I think my teeth are getting worse despite my daily brushing (D)
9	I brush each of my teeth carefully (A)
10	I have never been professionally taught how to brush (D)
11	I think I can clean my teeth without using toothpaste (A)
12	I often check my teeth in a mirror after brushing (A)
13	I worry about having bad breath
14	It is impossible to prevent gum disease with toothbrushing alone (D)
15	I put off going to the dentist until I have a toothache (D)
16	I have used a dye to see how clean my teeth are (A)
17	I use a toothbrush which has hard bristles
18	I don’t feel I’ve brushed well unless I brush with strong strokes
19	I feel I sometimes take too much time to brush my teeth (A)
20	I have had my dentist tell me that I brush very well
21	I think oral hygiene is important for general body health
22	Practical and theoretical courses at the dental school helped me learn how to perform my own oral hygiene
23	I brush my teeth two or more times a day
24	I floss regularly every day
25	I think my teeth are a problem for me
26	I smoke at least once a week
27	I drink alcohol at least once a week
28	I find that I use my phone/computer for longer than I planned
29	I go to the dentist for regular check-ups at least once a year

Before the questionnaire was administered, the purpose of the questionnaire and how to fill it out were explained to the students. If they volunteered, they were allowed to participate in the study after written consent was obtained. The students were asked to fill in the forms themselves, and the students who answered the questions completely were included in the study.

Ethical considerations

Ethical approval for the study was obtained from the local ethics committee of Alanya Alaaddin Keykubat University (approval number: 2022/11-04).

Statistical analysis

Statistical analyses of the obtained data were performed using SPSS Statistics version 22 software (IBM Corp., Armonk, NY, USA). Shapiro-Wilk test was used to evaluate the normality of the data. The chi-square test was used to analyze the categorical data of the students’ agree responses according to their academic level, and the Mann-Whitney U test was used to compare the mean HU-DBI scores according to academic level and gender because the data did not show normal distribution. The statistical significance level was set at p-values <0.05.

## Results

The mean age of the 250 students included in the study was 20.96 ± 1.66 years. Table 2 shows the mean age and mean HU-DBI score of students by academic year. In total, 157 (62.8%) were preclinical and 93 (37.2%) were clinical students.

**Table 2:** Mean age and HU-DBI scores of students by academic years. HU-DBI: Hiroshima University Dental Behavior Inventory

Academic years	N (%)	Mean age (years) (SD)	Mean HU-DBI scores (SD)
1	52 (20.8%)	19.15 (0.98)	5.21 (1.72)
2	51 (20.4%)	20.20 (1.33)	6.12 (1.39)
3	54 (21.6%)	21.28 (1.46)	5.69 (1.53)
4	61 (24.4%)	21.80 (0.73)	5.90 (1.52)
5	32 (12.80%)	23.00 (0.88)	7.41 (1.43)
Total	250 (100%)	20.96 (1.66)	5.95 (1.65)

Table 3 shows the mean HU-DBI scores of students according to their academic levels and gender. The mean HU-DBI score for the entire student population was 5.95 ± 1.65. While the mean HU-DBI score of preclinical students was 5.67 ± 1.59, the mean HU-DBI score of clinical students was 6.42 ± 1.65, and this difference was statistically significant (p < 0.001). The mean HU-DBI score of male and female students was 5.63 ± 1.55 and 6.24 ± 1.69, respectively, and this difference was statistically significant (p = 0.007).

**Table 3:** HU-DBI scores according to students’ academic level and gender. *Mann-Whitney U test. HU-DBI: Hiroshima University Dental Behavior Inventory

Parameters	N (%)	Mean HU-DBI scores (SD)	P-value*
Academic level			
Preclinical	157 (62.8%)	5.67 (1.59)	<0.001
Clinical	93 (37.2%)	6.42 (1.65)	
Gender			
Male	119 (47.6%)	5.63 (1.55)	0.007
Female	131 (52.4%)	6.24 (1.69)	
Total	250 (100%)	5.95 (1.65)	

Table 4 shows the percentages of the agree responses according to students’ academic level. When the preclinical students were compared with the clinical students, statistically significantly more preclinical students reported that their gums bled, they were worried about the color of their teeth, they would not be able to avoid having false teeth in their old age, they used a toothbrush with hard bristles, and they felt that their teeth were not cleaned completely if they did not brush with hard strokes (p < 0.05). Clinical students were statistically significantly more likely than preclinical students to report that they brushed each tooth carefully, that their dentist told them that they brushed their teeth very well, and that they went for regular dental check-ups at least once a year (p < 0.05).

**Table 4:** Numbers and percentages of agree responses according to students’ academic level. *: chi-square test.

Item number	Preclinical N (%)	Clinical N (%)	Total N (%)	P-value*
1	127 (80.9%)	69 (74.2%)	196 (78.4%)	0.214
2	32 (20.4%)	7 (7.5%)	39 (15.6%)	0.007
3	73 (46.5%)	31 (33.3%)	104 (41.6%)	0.041
4	31 (19.7%)	13 (14%)	44 (17.6%)	0.247
5	15 (9.6%)	11 (11.8%)	26 (10.4%)	0.569
6	26 (16.6%)	6 (6.5%)	32 (12.8%)	0.021
7	22 (14%)	10 (10.8%)	32 (12.8%)	0.456
8	37 (23.6%)	15 (16.1%)	52 (20.8%)	0.161
9	104 (66.2%)	76 (81.7%)	180 (72%)	0.008
10	61 (38.9%)	29 (31.2%)	90 (36%)	0.222
11	19 (12.1%)	8 (8.6%)	27 (10.8%)	0.389
12	137 (87.3%)	88 (94.6%)	225 (90%)	0.061
13	103 (65.6%)	67 (72%)	170 (68%)	0.292
14	124 (79%)	65 (69.9%)	75.6 (189%)	0.106
15	97 (61.8%)	47 (50.5%)	144 (57.6%)	0.082
16	4 (2.5%)	0 (0%)	4 (1.6%)	0.121
17	41 (26.1%)	12 (12.9%)	53 (21.2%)	0.014
18	61 (38.9%)	23 (24.7%)	84 (33.6%)	0.022
19	30 (19.1%)	23 (24.7%)	53 (21.2%)	0.293
20	44 (28%)	38 (40.9%)	82 (32.8%)	0.037
21	154 (98.1%)	91 (97.8%)	245 (98%)	0.896
22	111 (70.7%)	76 (81.7%)	187 (74.8%)	0.052
23	124 (79%)	82 (88.2%)	206 (82.4%)	0.065
24	22 (14%)	19 (20.4%)	41 (16.4%)	0.185
25	48 (30.6%)	22 (23.7%)	70 (28%)	0.239
26	53 (33.8%)	41 (44.1%)	94 (37.6%)	0.103
27	43 (27.4%)	35 (37.6%)	78 (31.2%)	0.091
28	126 (80.3%)	82 (88.2%)	208 (83.2%)	0.106
29	66 (42%)	54 (58.1%)	120 (48%)	0.014

## Discussion

The HU-DBI is an effective tool for defining variations in oral health attitudes between dental students in various countries because of differences in the education systems and curricular differences of dental faculties [9,16]. This can serve as a foundation for the development of supplementary programs aimed at enhancing the attitudes and behaviors of dental students [24]. The Turkish version of the HU-DBI, which had been previously translated by Camgöz and Gürgan in stages in accordance with the literature, was used [23]. To determine attitudes and behavioral differences in oral health, nine additional items were included in the questionnaire by examining the questions in the modified versions.

The results of this study were consistent with the studies conducted by Peker et al. (2010) and Yildiz and Dogan (2011), in which oral health attitudes and behaviors of dental students were evaluated with the HU-DBI questionnaire [6,20]. Furthermore, studies by Al-Wesabi et al. (2019) in Egypt, Pacauskiene et al. (2014) in Lithuania, and Dumitrescu et al. (2007) in Romania, which assessed dental students’ oral health attitudes and behaviors with the HU-DBI questionnaire, have consistently shown that clinical students tend to achieve higher scores on the HU-DBI assessment compared to their preclinical counterparts [11,25,26]. In contrast to our findings, studies conducted among dental students by Dagli et al. (2008) in India and Riad et al. (2022) in Estonia revealed no significant difference in the HU-DBI scores between clinical and preclinical students [7,12]. The prevailing thought regarding the difference proposes that dental education enhances students’ understanding of oral health, potentially leading to more favorable attitudes and behaviors among students [11].

In this study, the mean HU-DBI score for Turkish dental students was 5.95 ± 1.65, which is comparable to the findings of a previous study by Yildiz and Dogan in 2011 (6.53 ± 1.99), which evaluated oral health attitudes and behaviors in Turkish dental students using the HU-DBI questionnaire [20]. However, it was found to be higher than the average HU-DBI score (5.07) reported for dental students in China [17]. Previous research findings indicated that the average HU-DBI score among dental students in different countries was as follows, in order from high to low: Switzerland [13] 8.02 ± 1.27, Finland [14] 7.15 ± 1.13, Japan [14] 6.64 ± 2.47, Croatia [27] 6.62 ± 1.54, and Lithuania [25] 6.35 ± 1.43. Kawamura et al. (2005) noted that cultural orientations can yield valuable results in transnational comparisons of oral health attitudes and behaviors, especially among dental students [9].

The mean HU-DBI score for male subjects was 5.63 ± 1.55 and for female subjects was 6.24 ± 1.69, and this difference was statistically significant (p < 0.05). The effect of gender has not been evaluated in previous studies on this subject in the Turkish population. Therefore, our study is the first to examine the effect of gender on HU-DBI scores by evaluating attitudes and behaviors related to oral health in dental students in the Turkish population. Gender was a factor influencing the mean HU-DBI scores in our study. This finding is consistent with previous survey studies comparing oral health-related HU-DBI scores between genders among dental students in Jordan in 2004 by Al-Wahadni et al. [28], in the United Arab Emirates in 2013 by Rahman and Al Kawas [21], and in Sudan in 2015 by Al-Shiekh et al. [29]. This may be attributed to the fact that women are generally more aesthetically conscious and are likely to have more positive self-care attitudes to improve their appearance and self-esteem [29,30].

There was a statistical difference between clinical and preclinical students in the mean scores for “My gums tend to bleed when I brush my teeth” (item 2), “I brush each of my teeth carefully” (item 9), “I use a toothbrush which has hard bristles” (item 17), “I don’t feel I’ve brushed well unless I brush with strong strokes” (item 18), and “I have had my dentist tell me that I brush very well” (item 20). This could indicate an enhanced understanding of periodontal disease prevention as a result of the developed practical knowledge of clinical students. The results of this study were consistent with the studies conducted by Peker et al. (2010) and Yildiz and Dogan (2011), in which oral health attitudes and behaviors of dental students were evaluated with the HU-DBI questionnaire [6,20].

A lower percentage of clinical students expressed agreement with “I think that I cannot help having false teeth when I am old” in item 6 than preclinical students. This may be attributed to the fact that as the level of education increases, students pay more attention to their teeth and are aware of the negative impact of tooth loss on dental functions and aesthetics [28]. Similar results were observed in studies conducted in Egypt [11], the United Arab Emirates [21], and Turkey [6,20].

The percentage of agree responses in item 3, “I worry about the color of my teeth,” was 46.5% in preclinical students, and this rate was higher than in clinical students. The perception of tooth color is subjective and can be influenced by cultural norms, aesthetic considerations, and the extent of oral health education [10]. This result was consistent with the study conducted by Yildiz and Dogan (2011), in which oral health attitudes and behaviors of dental students were evaluated using the HU-DBI questionnaire in the Turkish population [20].

The strengths of this study include the pretesting process while developing the questionnaire items, investigating the relationship between gender and HU-DBI scores among dental students, and ensuring participation from all academic years.

The study provided information on undergraduate dental students’ oral health attitudes and behaviors, but there are some limitations. The study focused on perspectives on the impact of preclinical and clinical student status on self-care practices, but the educational curriculum was not investigated in detail. As this study did not clinically measure the oral health status of students and was based on self-report, students may have over or underreported their oral health practices.

## Conclusions

The results showed that undergraduate clinical dentistry students generally had better attitudes and behaviors of oral health self-care than preclinical students. In addition, female students presented higher mean HU-DBI scores compared to male students. Appropriately designed oral health education programs may be beneficial to improve preclinical dental students’ oral health awareness. Further studies are needed to investigate the detailed oral health status of dental students clinically and to evaluate the relationship with their oral health attitudes and behaviors.
